# Determinants of Heterosexual Adolescents Having Sex with Female Sex Workers in Singapore

**DOI:** 10.1371/journal.pone.0147110

**Published:** 2016-01-25

**Authors:** Junice Y. S. Ng, Mee-Lian Wong

**Affiliations:** Saw Swee Hock School of Public Health, National University of Singapore, Singapore, Singapore; David Geffen School of Medicine at UCLA, UNITED STATES

## Abstract

**Objectives:**

We assessed the proportion of and socio-ecological factors associated with ever having had sex with female sex workers (FSWs) among heterosexual adolescents. We also described the characteristics of the adolescents who reported inconsistent condom use with FSWs.

**Methods:**

This is a cross-sectional study (response rate: 73%) of 300 heterosexually active male adolescents of 16 to 19 years attending a national STI clinic in Singapore between 2009 and 2014. We assessed the ecological factors (individual, parental, peer, school and medial influences) and sexual risk behaviors using a self-reported questionnaire. Poisson regression was used to obtain the adjusted prevalence ratios (aPR) and confidence intervals (CI).

**Results:**

The proportion of heterosexual male adolescents who had ever had sex with FSWs was 39%. Multivariate analysis showed that significant factors associated with ever having had sex with FSWs were sex initiation before 16 years old (aPR 1.79 CI: 1.30–2.46), never had a sexually active girlfriend (aPR 1.75 CI 1.28–2.38), reported lower self-esteem score (aPR 0.96 CI: 0.93–0.98), higher rebelliousness score (aPR 1.03 CI: 1.00–1.07) and more frequent viewing of pornography (aPR 1.47 CI: 1.04–2.09). Lifetime inconsistent condom use with FSWs was 30%.

**Conclusions:**

A significant proportion of heterosexual male adolescents attending the public STI clinic had ever had sex with FSWs. A targeted intervention that addresses different levels of influence to this behavior is needed. This is even more so because a considerable proportion of adolescents reported inconsistent condom use with FSWs, who may serve as a bridge of STI transmission to the community. National surveys on adolescent health should include the assessment of frequency of commercial sex visits and condom use with FSWs for long-term monitoring and surveillance.

## Introduction

Clients of female sex workers (FSWs) are the largest core group in the transmission of sexually transmitted infections (STIs) and human immunodeficiency virus (HIV) in Asia. [1] In China, clients of FSWs were found to have 12 times and 6 times the odds of being infected with HIV and syphilis respectively compared to the general adult population. [2] Despite the risks, patronage of commercial sex is viewed as a social activity in Asia. [3, 4]

A national survey of 46,961 sexually active males in India reported that younger males have higher propensity to buy sexual services compared to adults. [5] In this study, 15 to 24 year olds were twice more likely than those aged 45 and above to have engaged in sex with FSWs in the past one year. Of those between 15 to 24 years old, 41% did not use condoms consistently with FSWs. However, the proportion of adolescents, defined as 10 to 19 years old by World Health Organization, who engaged in sex with FSWs and the factors associated with this behavior were not reported in this study. To the best of our knowledge, there is currently no specific intervention targeting this behavior specifically for adolescents in Asia probably because resources are channeled to interventions targeting adult men and FSWs. Another possible reason could be due to the paucity of data on the magnitude of this behavior and its associated factors, which could be ascribed to the legal and ethical restrictions such as parental consent for the collection of this sensitive data. [6] Nonetheless, targeting this group is of paramount importance because it can influence their sexual behaviors positively during young adulthood. [7] Furthermore, Asia has the second largest number of new HIV infections among older adolescents. [8] Yet, there is a lack of progress in preventing new HIV infections for this group due to insufficient data. [6]

While most studies on correlates associated with purchasing sex include male adult samples [2, 5, 9–11], few looked into the associations specifically among adolescents. [12, 13] Findings on the influence of sexual abuse and risk behaviors on purchasing sex among adolescents have been mixed too. A national study in United States evaluated the factors in early adolescence (aged 12 to 17 years) that were associated with buying sex in later years (aged 18 to 26 years). [13] It was found that history of sexual abuse, drug use and ever running away from home were risk factors. On the contrary, in a school-based survey in Canada, sexual abuse and drug use were not associated with buying sexual services. In this study, 3% of those between 15 to 18 years old had ever bought sex and they were more likely to have observed sexualized social activities and also have more approving attitude towards prostitution. [12] While this study aims to enhance the understanding on the factors associated with buying sexual services among adolescents, we would also like to fill the gap in the information on sexual risk behaviors associated with having sex with FSWs. This leads to the primary objective of this study, which is to describe the proportion of sexually active male adolescents visiting the public STI clinic in Singapore who had ever had sex with FSWs, as well as the socio-ecological factors and sexual behaviors associated with this behavior. The secondary objective is to describe the characteristics of adolescents who did not use condoms consistently with FSWs. The findings would help inform programs for sexually active male adolescents, which is made even more critical in Singapore as adolescents are being excluded from national behavioral surveillance on sexual practices.

## Materials and Methods

### Participants and recruitment

Data for this analysis were drawn from the baseline needs assessment for a sexual health intervention (registered with ClinicalTrials.gov, number NCT02461940) for adolescents attending the Department of STI Control (DSC) clinic, the only national STI clinic in Singapore. Data were collected between November 2009 and December 2014. The reason for conducting the study in this clinic is twofold. Firstly, approximately 80% of the adolescents with notifiable STIs in Singapore attended this clinic annually. [14] Secondly, these adolescent attendees are also the core group of STI transmission among the adolescents in Singapore. Inclusion criteria for this study were: never married male adolescents who reported being exclusively heterosexual, defined as having intercourse with a female, and aged 16 to 19 attending the clinic for the first time. As the legal age of sex in Singapore is 16 years old, we did not recruit adolescents below 16 years old, who had to be placed under police investigation for statutory rape.

This study was approved by the National Healthcare Group Domain Specific Review Board. Adolescents who took part in the study signed the consent form after receiving an explanation of the study, reading the information sheet and clarifying questions with the interviewer. Signed consent forms were kept in a locked cabinet in DSC clinic, rather than returned to the participants. This was because, given the nature of our study and the age of the participants, they were unlikely to bring home the forms which indicated their visit to DSC clinic. Instead, they might discard the signed forms which bore their names and identity numbers in public places. Therefore, it was not advisable to return the signed forms to the participants. Nevertheless, the signed consent form was made available to the participant at the clinic should the participant want to access it. As part of the study, cost for STI laboratory tests (up to US$50) was waived for enrolled participants.

There were two parts to the questionnaire. The first part on demographics and parenting was administered face-to-face by the local staff with study participants in a private area of the clinic. The second part, which was self-administered, consisted of questions of sensitive nature such as risk and sexual behaviors and was placed at the end of the survey. To reduce social desirability bias, participants were assured confidentiality and anonymity. They were informed about the study intent, which was to better understand their behaviors and to use the findings to plan programs for them.

Of the 409 eligible adolescents who visited the clinic during the study period, 300 (73%) consented and completed the baseline survey. The main reason for non-consent was the inability to commit to the sexual health interventional study. There were no differences between respondents and non-respondents in terms of age (p = 0.320) and ethnicity (p = 0.704).

### Measures

#### Outcome variable

This was a dichotomized variable of ever having had sex with FSWs, which was based on the question: “How many times did you have sex with a prostitute since you first had sex?” Those who answered “1” or more were categorized as “ever having had sex with FSWs” while those who indicated “0” were categorized as “never had sex with FSW”.

#### Factors associated with engaging in sex with FSWs

We adapted the multi-ecological model [15] to assess the factors associated with ever having had sex with FSWs, which included constructs that measured individual, parental, peer, school and media influences. For item-based constructs, each item was assessed using a Likert scale and the items were summed to create a score.

***Individual level***: We assessed the socio-demographic characteristics (age, type of residence, ethnicity, religion, schooling and working status and educational level), risk behaviors, history of sexual abuse and personality traits. Risk behaviors included smoking, alcohol drinking, gang fighting and drug use. The personality traits included the following: 7-item rebelliousness (Cronbach’s alpha = 0.62) [16], 6-item sensation-seeking (Cronbach’s alpha = 0.78) [16], 4-item perceived external control (Cronbach’s alpha = 0.72) [17] and 10-item Rosenberg self-esteem scale (Cronbach’s alpha = 0.66) [18]. Each item in the personality trait construct was assessed using a 4-point Likert scale of “Not like me”, “Sort of like me”, “A lot like me” and “Just like me”.

***Parental level***: Parental influence was assessed using the 7-item demanding parenting (Cronbach’s alpha = 0.79) and the 8-item authoritative parenting (Cronbach’s alpha = 0.72) indices [19] with responses for each item rated on a 4-point Likert scale, as well as the statement “I feel that I can go to parent/s with questions about sex”.

***Peer level***: Peer influence was assessed based on two components: the 6-item peer connectedness using a 4-point Likert scale (Cronbach’s alpha = 0.74) [20] and the question “How much pressure is there from your friends for you to have sexual intercourse?”

***School level***: *S*chool performance was assessed by the questions “Where would you rank yourself in academic performance in school?” and “Where would you rank yourself in co-curricular activities in school?”

***Media level***: Sexual content in the mass media was assessed based on 3 types of exposure: 1) public-access media, 2) banned media in Singapore, that is, pornographic material and 3) informational media. Public-access media refers to TV programs/movies/videos/songs depicting sex or sexual scenes. Frequency of exposure was assessed using a 3-item composite score on sexual media, [21] with each item on a 4-point scale (hardly, once in a while, quite often, almost every time). Exposure to pornography was determined by asking, “How often do you read or watch pornographic material?” Exposure to informational media refers to ever having read or watched TV programs/movies about someone infected with STIs/HIV/AIDS. An example of the yes/no statement is “I have read in the newspaper or magazine about someone who is infected with sexually transmitted diseases.”

#### Sexual behaviors

Participants reported the age of first sex (which was defined as oral, vaginal or anal sex) and we defined early sexual debut as below 16 years old. Overall lifetime condom use for vaginal sex with all partners was based on “Have you or your partner ever used a condom for vaginal sex?” with the options of “Always”, “Sometimes”, “Not at all” and “Cannot remember”. The same question and options were applied to condom use for oral and anal sex. In addition, they were asked to indicate their first sexual partner with the options of “girlfriend, prostitute, client, casual partner or others”. Participants were required to indicate the number of sexual partners in the lifetime and also the number for each of the following type of partners, “Girlfriend(s), Prostitute(s), Client(s), Stranger/Acquaintance and Others”.

Diagnosed STIs were confirmed with laboratory tests at the time of enrolment to the study. These included infectious syphilis, (cervical, urethral, pharyngeal, rectal) gonorrhea, chlamydia, non-gonococcal urethritis, genital herpes, genital warts, molluscum contagiosum, pubic lice and HIV.

#### Attitudes and perceptions

Attitude towards condom use was based on the summed score of 7 statements. Each statement was assessed with a 5-point Likert scale of “Strongly disagree”, “Disagree”, “Neutral”, “Agree” and “Strongly agree”. These included: (1) Condom is an effective way of protecting against STIs. (2) Condoms break easily. (3) I find it bothersome/inconvenient to use condoms. (4) Condoms reduce sexual pleasure. (5) Condoms make sex less messy. (6) Condoms are expensive. (7) It is convenient/easy to get a condom when I need one. Items 2, 3, 4 and 6 were reverse coded. We used “What do you think is your chance of getting STIs?” to assess their perceived chance of getting STIs. Participants were also asked to select the statement that best describes how they feel about sexual intercourse before marriage.

#### Commercial sex visits and condom use

Respondents who reported ever having had sex with FSWs were also asked to provide more information about this behavior. Consistent condom use with FSWs was assessed using “Have you ever thought of using condoms with prostitutes in the last 1 year?” The option “I use condoms all the time with prostitutes” was considered as consistent condom use and the rest of the options “I never thought about using it”, “I have thought about it but have not started using it yet”, “I want to use condoms but I don’t know how”, “I have used condoms before but not now” and “I have been using condoms sometimes” were grouped as inconsistent condom use. They were also asked, with the option of choosing more than one response, the country (that is, Singapore, Thailand, Indonesia, Cambodia, Malaysia, China, other Asian countries or West) and the type (that is, brothel, streets, massage parlors, bars/pubs or hotels) of commercial sex visits. We classified streets, massage parlors, bars/pubs and hotels as non-brothel-based settings.

### Statistical analyses

In bivariate analysis, categorical variables were assessed using chi-square or trend tests, whereas continuous variables were assessed using Wilcoxon rank-sum test. For multivariate analysis, Poisson regression with robust variance was used instead of logistic regression due to the high proportion (>10%) of adolescents reporting ever having had sex with FSWs. We used forward stepwise method to construct the model. Each theoretically plausible independent variable with p<0.1 from the bivariate analysis was entered into the model, using forward selection. These included alcohol consumption, rebelliousness, self-esteem, perceived external control, academic performance, co-curricular activities, pornography viewing, age of first sex before 16 years old and ever had sexually active girlfriend. The first variable that accounted for maximum variation in the model was selected, and the second variable was likewise chosen. Subsequent variables were added until there was no significant variation in the prediction of the outcome variable to obtain the most parsimonious model. The model was adjusted for demographic variables (that is, age, ethnicity, type of residence, education level) and year of recruitment. The goodness-of-fit for the final model indicated that the model fitted the data well (p = 1.00). Statistical significance was set at p<0.05 and adjusted prevalence ratios (aPR) were reported. We used the software package Stata 14.0 (Stata Corp, College Station, Tex) to perform the statistical analyses.

## Results

### Demographic characteristics and sexual behavior

Overall, the median age of participants was 18 years (interquartile range [IQR]: 18–19). Slightly more than half (57%) were Chinese, 33% were Malay and the rest were Indian and Eurasian. There were no statistically significant interaction terms between ethnicity and the independent variables for ever having had sex with FSWs. Forty-seven percent of the participants were not schooling. About 40% of the adolescents had ≤10 years of schooling. Of the 140 who were not schooling at the point of survey, 66 (47%) were school dropouts. The socio-demographics and risk behaviors are summarized in Table 1. The median first sex age was 16 years old (IQR: 15–18) and the median number of sex partners in the lifetime was 3 (IQR: 2–6). None of the participants was ever paid for sex. Forty-seven percent were positive with STIs. There were no cases of diagnosed HIV.

**Table 1:** Sex with female sex worker by selected characteristics among sexually active adolescents aged 16–19.

		n (%)		
Factors	Total (N = 300)	Ever had (n = 118)	Never had (n = 182)	p-value
**Age at recruitment** (Median (IQR))	300	19 (18–19)	18 (17.5–19)	0.16
<18 years	67	22 (18.6)	45 (24.7)	0.26
≥18 years	233	96 (81.4)	137 (75.3)	
**Ethnicity**				
Malay	99	29 (24.6)	70 (38.5)	0.02
Non-Malay^a^	201	89 (75.4)	112 (61.5)	
**Religion**				
No religion	65	27 (22.9)	38 (20.9)	0.08
Islam	101	31 (26.3)	70 (38.5)	
Non-Islam^b^	134	60 (50.8)	74 (40.7)	
**Type of residence**^c^				
HDB 1/2-rooms	22	5 (4.2)	17 (9.4)	0.13
HDB 3-rooms	57	27 (22.9)	30 (16.6)	
HDB 4/5-rooms/Private residence	220	86 (72.9)	134 (74.0)	
**Currently schooling**				
Yes	159	60 (51.3)	99 (54.4)	0.64
No	140	57 (48.7)	83 (45.6)	
**Current/Highest education level**				
Primary/Secondary	117	45 (39.1)	72 (39.8)	0.62
Junior College/Technical Diploma/University	89	38 (33.0)	51 (28.2)	
Institute of Technical Education	90	32 (27.8)	58 (32.0)	
**Currently working**				
No	126	46 (39.0)	80 (44.0)	0.45
Yes	99	38 (32.2)	61 (33.5)	
National Service^d^	75	34 (28.8)	41 (22.5)	
**Smoking**				
Never-smoker/Ex-smoker/Tried once or twice	78	32 (27.1)	46 (25.4)	0.27
Social smoker	54	26 (22.0)	28 (15.5)	
Regular smoker	167	60 (50.9)	107 (59.1)	
**Alcohol drinking**				
Never	65	19 (16.1)	46 (25.3)	0.02^
Less than once a month	141	54 (45.8)	87 (47.8)	
More than once a month	94	45 (38.1)	49 (26.9)	
**Involved in gang fights**				
Ever	63	27 (22.9)	36 (19.8)	0.56
Never	237	91 (77.1)	146 (80.2)	
**Experimented with drugs**				
Ever	41	21 (17.9)	20 (11.1)	0.12
Never	256	96 (82.1)	160 (88.9)	
**History of sexual abuse**				
Yes	5	2 (1.7)	3 (1.6)	1.00
No	295	116 (98.3)	179 (98.4)	
**Personality** (Median (IQR))				
Rebelliousness score^e^	294	14 (11–17)	12 (10–16)	0.00
Rosenberg self-esteem score^f^	293	27 (24–31)	29 (25–32)	0.02
Perceived external control score^g^	298	10 (8–11)	9 (7–11)	0.01
Sensation-seeking score^h^	290	13 (11–15)	13 (10–15)	0.24
**Parenting Influences**				
**Demanding parenting score**	290	14 (10–18)	15 (11–19)	0.20
**Authoritative parenting score**	290	20 (17–23)	20 (17–24)	0.49
**I feel that I can go to my parents with questions about sex**				
Disagree/Strongly disagree	161	67 (59.3)	50 (27.8)	0.46
Agree/Strongly agree	75	25 (22.1)	94 (52.2)	
Not sure	57	21 (18.6)	36 (20.0)	
**Peer influences**				
**Peer connectedness score**	296	17 (15–20)	17 (15–20)	0.85
**Peer pressure to have sex**				
No pressure at all	181	67 (56.8)	114 (63.3)	0.31^
A little pressure	80	35 (29.7)	45 (25.0)	0.53
Moderate /A lot pressure	37	16 (13.6)	21 (11.7)	
**School environment**				
**Academic performance**^i^				
Above average	76	21 (18.8)	55 (31.4)	0.02
Average or below	211	91 (81.2)	120 (68.6)	
**Co-curricular activities performance**^i^				
Above average	134	43 (39.4)	91 (54.2)	0.02
Average or below	143	66 (60.6)	77 (45.8)	
**Media influences**				
**Sexual media score**^j^ (Median (IQR))	298	7 (6–8)	7 (5–8)	0.13
**Frequency of viewing pornography**				
Whenever I have time	42	23 (19.5)	19 (10.4)	<0.001^
Once in a while	144	66 (55.9)	78 (42.9)	<0.001
Hardly	101	28 (23.7)	73 (40.1)	
Never before	13	1 (0.9)	12 (6.6)	
**Read or watched television/movies about persons with STIs/HIV/AIDS**				
Ever	98	37 (31.4)	61 (33.5)	0.71
Never	202	81 (68.6)	121 (66.5)	

^Indicates chi trend.

^a^ Comprise 171 Chinese, 28 Indian and 2 Eurasian males.

^b^ Comprise 69 Buddhist, 32 Christian, 15 Hindu, 8 Taoist, 8 Catholic, 2 Sikh males.

^c^ The different room types are used as a proxy indicator of socioeconomic status. HDB refers to Housing Development Board apartments (public housing flats). More than 80% Singaporeans live in HDB flats.

^d^ A two-year conscription for all Singaporean males who have reached the age of 18.

^e^ A higher score represents more rebelliousness.

^f^ A higher score represents higher self-esteem.

^g^ A higher score represents higher perceived external control.

^h^ A higher score represents more sensation-seeking.

^i^ *Above average* includes “Top 10%, “Just below 10% to just above average.” *Average or below* includes “Average”, “Below Average” and “Bottom or near the bottom.”

^j^ A higher score represents greater exposure to public-access media with sexual content.

This is based on the summed scores of the following 3 questions, with each assessed using a 4-point scale. (1) Based on television programs and movies you have watched, how often do you hear people talking about having sex? (2) Based on television programs and movies you have watched, how often do you see people kissing, touching, or undressing themselves? (3) Based on popular songs you have listened to, how often do you hear people talking about having sex?

### Proportion of heterosexual male adolescents who had sex with FSWs and factors associated with ever having had sex with FSWs

One hundred and eighteen (39%, 95% CI: 34%-45%) reported ever having had sex with FSWs, with significantly more Chinese (44%) than Malays (29%) (p = 0.02). In the bivariate analyses, those who reported higher rebelliousness (p = 0.002), lower self-esteem (p = 0.02) and higher perceived external control (p = 0.01) scores, and rated their academic performance as average or below (p = 0.02) were more likely to have had sex with FSWs. Higher frequency of viewing pornography (p<0.001) was significantly more common among those who had ever had sex with FSWs. Educational level (p = 0.62), parental influence [Demanding parenting index: p = 0.20; Authoritative index: p = 0.49] and peer influence [Peer connectedness: p = 0.85] were not associated with this behavior.

As shown in Table 2, males who had ever had sex with FSWs were more likely to have sex before 16 years of age (p = 0.01) and have more sexual partners (p<0.001). However, they were significantly less likely to have ever had a sexually active girlfriend (p<0.001) and more likely to report lifetime consistent condom use with all partners for vaginal (p<0.001), oral (p<0.001) and anal sex (p = 0.048) with all types of partners.

**Table 2:** Sex with female sex worker by sexual behaviors among sexually active adolescents aged 16–19.

		n (%)		
Factors	Total (N = 300)	Ever had (n = 118)	Never had (n = 182)	p-value
**Perceptions and attitudes**				
**Attitude score towards condom use**^a^ (Median (IQR))	297	23 (21–25)	22 (21–24)	0.08
**Perceived chance of getting STIs**				
Not at all/Very unlikely <25%	123	47 (44.3)	76 (52.8)	0.20
Some chance 25–75%/High Chance >75%	127	59 (55.7)	68 (47.2)	
**Perception towards premarital sex**				
OK even if couple is not in love	59	29 (24.8)	30 (16.6)	0.10
OK if couple is in love/ planning to get married/Not OK	239	88 (75.2)	151 (83.4)	
**Sexual behaviors**				
**First sex age** (Median (IQR))	297	16 (15–18)	16 (15–18)	0.11
<16 years	93	47 (39.8)	46 (25.7)	0.01
≥16 years	204	71 (60.2)	133 (74.3)	
**Number of sexual partners** (Median (IQR))	300	4.5 (2–9.25)	2 (1–5)	<0.001
**Ever had sexually active girlfriend**				
Yes	244	82 (69.5)	162 (89.0)	<0.001
No	56	36 (30.5)	20 (11.0)	
**Ever had oral sex**				
Yes	245	105 (92.9)	140 (78.7)	0.00
No	46	8 (7.1)	38 (21.3)	
**Ever had anal sex**				
Yes	61	21 (17.9)	40 (22.0)	0.46
No	238	96 (82.1)	142 (78.0)	
**Condom use for vaginal sex for all partners**				
Always	67	42 (36.8)	25 (14.5)	<0.001^
Sometimes	137	49 (43.0)	88 (50.9)	
Not at all	70	17 (14.9)	53 (30.6)	
Cannot remember	13	6 (5.3)	7 (4.0)	
**Condom use for oral sex for all partners**				
Always	18	15 (12.9)	3 (1.9)	<0.001^
Sometimes	84	53 (45.7)	31 (19.5)	
Not at all	155	41 (35.3)	114 (71.7)	
Cannot remember	18	7 (6.0)	11 (6.9)	
**Condom use for anal sex for all partners**				
Always	22	13 (33.3)	9 (15.3)	0.04^
Sometimes	15	5 (12.8)	10 (16.9)	
Not at all	50	16 (41.0)	34 (57.6)	
Cannot remember	11	5 (12.8)	6 (10.2)	

^Indicates chi trend, excluding the option “Cannot remember.”

^a^ A higher score represents better attitude towards condom use.

On multivariate analysis (Table 3), adolescents who initiated sex before 16 years old (aPR 1.79 CI: 1.30–2.46), never had a sexually active girlfriend (aPR 1.75 CI 1.28–2.38), reported lower self-esteem score (aPR 0.96 CI: 0.93–0.98), higher rebelliousness score (aPR 1.03 CI: 1.00–1.07) and viewed pornography more frequently (aPR 1.47 CI: 1.04–2.09) were more likely to engage in sex with FSWs.

**Table 3:** Poisson regression models.

	Crude		Adjusted^a^	
Variables	PR (CI)	p-value	PR (CI)	p-value
Age of sexual initiation				
<16 years old	1.61 (1.19–2.18)	0.002	1.79 (1.30–2.46)	<0.001
Never had a sexually active girlfriend	1.91 (1.44–2.55)	<0.001	1.75 (1.28–2.38)	<0.001
Rosenberg self-esteem score^b^	0.97 (0.94–1.00)	0.022	0.96 (0.93–0.98)	0.002
Pornography (ref: Never before/Hardly)				
Whenever I have time/Once in a while	1.62 (1.15–2.28)	0.006	1.47 (1.04–2.09)	0.030
Rebelliousness score^b^	1.02 (0.99–1.05)	0.249	1.03 (1.00–1.07)	0.047

^a^ Adjusted for age at recruitment, ethnicity, type of residence, education, year of recruitment.

^b^ Entered as continuous variable.

### Commercial sex visits, condom use and STIs

Among those who had ever had sex with FSWs, 38% reported having had their first sex with a FSW, while the rest were mainly with a girlfriend (41%) or a casual partner (14%). The median lifetime number of sexual encounters with FSWs was 2 (IQR: 1–3). The most common location of buying sexual services was in Singapore (51%), followed by Thailand (40%) and Indonesia (17%). Overall, 30% (n = 35) did not use condoms consistently with FSWs in the last one year. Half of the respondents (51%) had ever had sex with brothel-based FSWs and 35% with the street workers.

We found that Malay adolescents were significantly less likely to use condoms with FSWs compared to non-Malays (59% vs. 20%, p<0.001). There was no significant difference in the number of sexual encounters with FSWs between those who used condoms consistently with FSWs and those who did not (Median (IQR): 2 (1–3) vs. 2 (2–3), p = 0.54). The consistent condom users also did not differ from the inconsistent condom users in their attitude score towards condom use (Median (IQR): 23 (20–25) vs. 23 (21–25), p = 0.80).

**Fig 1:**
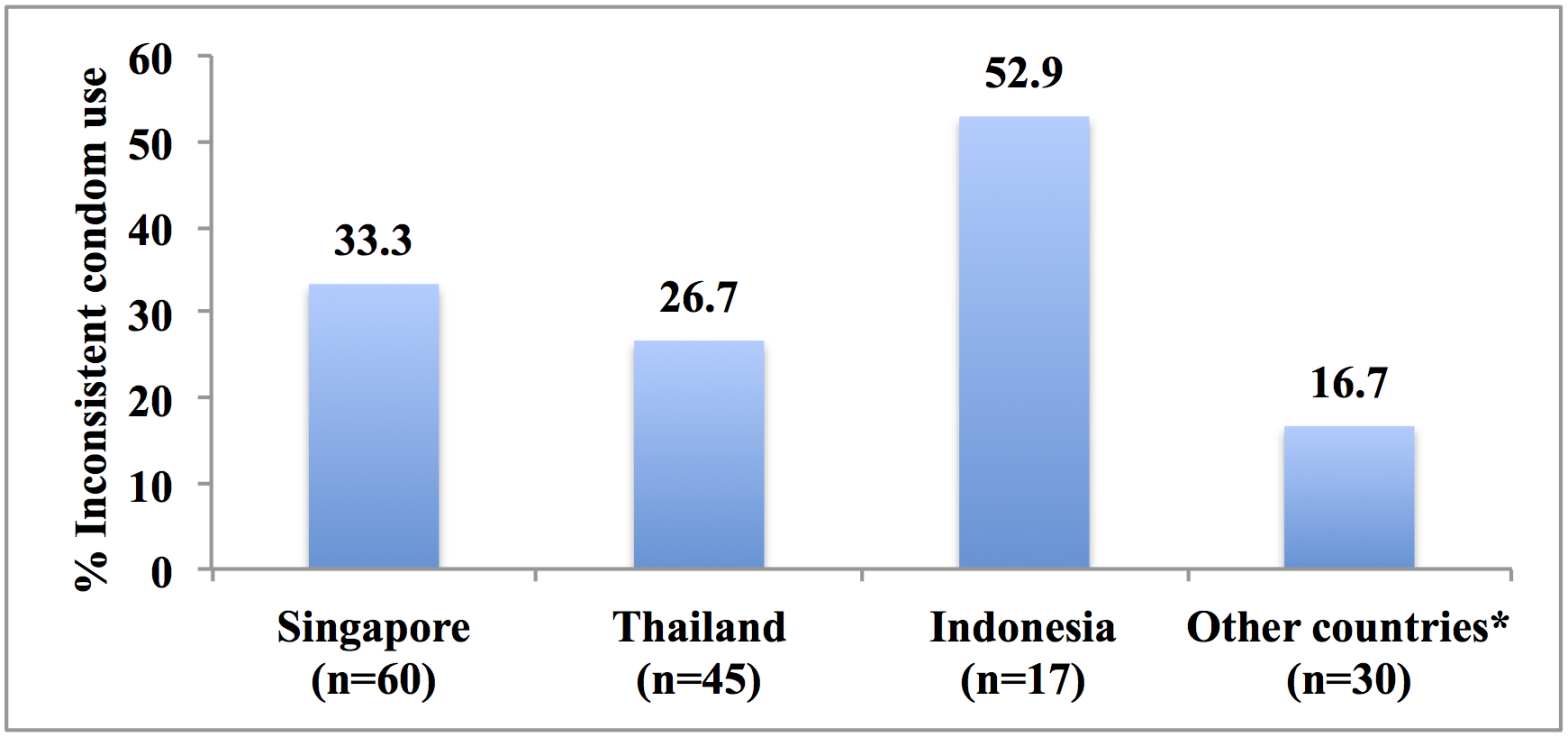
Percentage of sexually active adolescents aged 16–19 years who used condom inconsistently with female sex workers in the past year by country of female sex workers. * Comprise 10 China, 6 Malaysia, 2 Cambodia, 10 other Asian countries and 2 Western countries.

**Fig 2:**
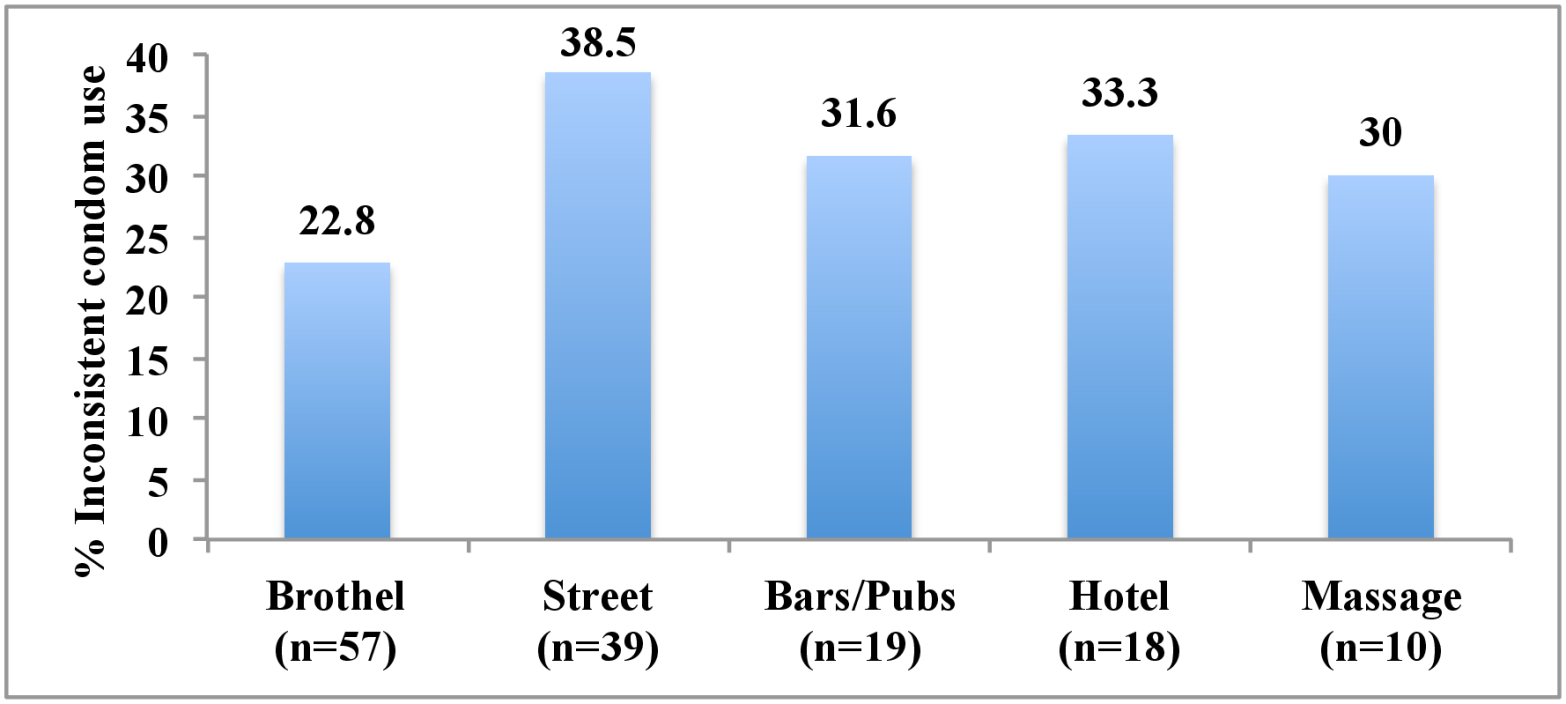
Percentage of sexually active adolescents aged 16–19 years who used condom inconsistently with female sex workers in the past year by type of female sex workers.

The proportion of diagnosed STIs at recruitment was found to be similar among those who engaged in sex with FSWs and those who did not (41.9% vs. 49.7%, p = 0.19). However, among adolescents who had ever had sex with FSWs, diagnosed STIs was significantly higher among those did not use condoms consistently with FSWs and all other partners compared to those who used condoms consistently (59% vs. 17%, p<0.001). Diagnosed STIs were also higher, although not statistically significant, among those who had ever had sex with non-brothel-based FSWs only compared to those who had ever had sex with brothel-based FSWs only (46% vs. 32%, p = 0.27). Figs 1 and 2 respectively show the percentage of those who used condoms inconsistently by the country and the type of FSWs. The highest rate of inconsistent condom use (53%) was reported among those who bought sexual services in Indonesia. Participants who engaged in sex with street workers reported the highest percentage of inconsistent condom use (39%) while those who had sex with brothel-based sex workers reported the lowest (23%).

## Discussion

A considerable proportion (39%) of heterosexually active adolescent males attending the public STI clinic in Singapore in our study reported ever having had sex with FSWs. This is lower than that reported in another study in STI clinics in Vietnam, in which 84% of adolescents aged 14 to 19 years had visited FSWs in the past one year. [9] We also found different levels of influence to this behavior. In the multivariate-adjusted model, we found that adolescents who reported younger age of sexual initiation, lower self-esteem score, higher rebelliousness score, never had a sexually active girlfriend, and more frequent viewing of pornography were more likely to report ever having had sex with FSWs.

As far as we know, there is only one study focusing on the behavior on engaging in sex with sex workers among adolescents and this was conducted among Canadian high school students aged 16 to 18. [12] This cross-sectional study looked into factors such as sexual precocity (that is 13 years or below), having casual partners, viewing online pornography and observing sexualized activities such as stripteases. After multivariate adjustment, only observing sexualized activities emerged to be a significant factor. This differed from our study findings, possibly due to different variable cutoffs and assessment methods. However, our results are consistent with studies on adult males engaging in commercial sex. A study on Spanish male population aged 18 to 49 found that those who were single and initiated sex before 16 years old were more likely to buy sex. [11] A report in Australia explained that adult males who are single lack the ability to socially interact in sexual situations and resort to FSWs for intimacy. [22] Additionally, viewing pornography was a significant factor associated with buying sexual services among Indian migrant workers with an average age of 27 years, and it was reasoned that pornography made them develop a more positive attitude towards paid sex. [23]

Our findings also demonstrated factors that are unique to adolescents, which is consistent with Jessor’s Problem Behavior Theory. [24] It explains that problem behaviors (such as engaging in sex with FSWs [5]) manifest during adolescence as a consequence of imbalance in control of the personality system (such as low self-esteem and rebelliousness), the perceived environment system (such as media and pornography) and behavior system (such as early age of sexual initiation). Of note, self-esteem was not associated with risky sexual behaviors (such as sexual debut and history of STIs) among adolescents in a systematic review. [25] However, it was a strong factor associated with ever having had sex with FSWs in our study. This could possibly be explained by them engaging in sex with FSWs as a means to boost their low self-esteem, which resulted from the inability to find a girlfriend. This is also congruent with a study finding among African American adolescents that found that they used sex to enhance their self-esteem or for bragging reasons. [26] Nevertheless, we will need to do further research to better understand the relationship between low self-esteem and buying sexual services. Interventions should not only target different levels of influence, but also address the problem behavior as a collective syndrome of problems rather than regarding them as being mutually exclusive.

We did not find diagnosed STIs to be associated with ever having had sex with FSWs. There are some possible explanations for this. Firstly, STIs were diagnosed at the point of enrolment to the study, whereas our study outcome was a lifetime prevalence of engaging in sex with FSWs. Therefore, participants who have bought sex before could have been positive with acute STIs and have it treated elsewhere prior to attending this clinic. Secondly, the risk of acquiring STIs from a FSW is also dependent on the condom usage with the sex worker and her STI status at that point of sexual contact. In fact, we found STIs to be significantly higher among those who did not use condoms consistently with FSWs. Lastly, about half of the participants bought sex from brothels in Singapore. All brothels in Singapore are licensed and 100% condom use has been established. In addition, brothel-based sex workers, under the Medical Surveillance Scheme, have to undergo bi-monthly screening for gonorrhea and chlamydia, and four-monthly screening for HIV and syphilis. Sex workers who are positive with STIs are treated at the clinic and have to stop sex work during the period of treatment.

There are limitations to this study which temper the generalizability to other populations. Firstly, even though this is the only specialist STI clinic in Singapore which attends to more than three quarters of notifiable STI cases among adolescents, it is only representative of sexually active adolescents who attend this clinic or have been referred to it for screening and treatment of STIs. Of note, not all sexually active adolescents have STI symptoms and therefore, adolescents who attend the STI clinic may not represent the sexually active adolescents in the general population. In addition, adolescents who engage in sex with FSWs may not have STIs and hence, may not seek medical care at the STI clinic. As the data was cross-sectional in nature, we could not establish the temporal relationship between the risk factors and ever having had sex with FSWs. Instead of lifetime condom use with FSWs, we only assessed condom use in the past year. We were also not able to draw any inferences on the motivations and the context of buying sex among adolescents, which justifies the need for qualitative research. Our relatively small sample size also limits our statistical power to assess independent factors associated with condom use with FSWs. Finally, the study findings cannot be generalized to male adolescents who reported engaging in sex with male partners or male sex workers. Nevertheless, our study has a high participation rate and a multi-ethnic sample. We have also applied an ecological model to systematically identify possible associations with this complex behavior. Most importantly, our findings provided insights on the behavior of buying sex among adolescents and its public health implications.

Our finding on the high proportion of buying sex among heterosexually active adolescents attending an STI clinic is of public health concern. About one third of adolescents also did not use condoms consistently with FSWs. They are a potential source of contracting and transmitting STIs to the general population such as their regular or casual partners, with whom they reported an even lower likelihood of condom use. [27] Furthermore, adolescents who had sex with non-regulated sex workers such as streetwalkers reported a higher proportion of inconsistent condom use compared to those who had sex with brothel-based sex workers in Singapore, where a 100% condom use program has been established. [28] It is also challenging to track Singaporean adolescents who buy sex from FSWs operating illegally on the streets or overseas. Current sex education in schools in Singapore may be reticent in educating adolescents about buying sex and condom use. Even then, school dropouts may not be able to benefit from this program.

Interventions targeting adolescent clients attending the only public STI clinic in Singapore serve as a practical and feasible strategy to provide STI-preventive education, screening and treatment to adolescents buying sex from these avenues, although we acknowledge that adolescents might seek health care from other settings. Another reason why interventions should start during adolescence is that adolescents are more amenable to behavioral change than adults. [29] Such behavioral interventions should be tailored to adolescents engaging in sex with FSWs by modifying different levels of influences such as individual and media-related factors. Given the findings from this survey in STI clinic, national surveys on sexual behaviors should include adolescents and incorporate questions on sexual encounters with FSWs to allow for long term monitoring and surveillance of this behavior. Future studies with a larger number of adolescents engaging in sex with FSWs could provide further insights into their condom-use behaviors.

## Conclusions

There is a notable proportion of male adolescents attending the STI clinic who reported engaging in sex with FSWs. As a significant proportion of them did not use condoms, they are a potential bridge for STI transmission to the general female population in Singapore and beyond. Therefore, targeted prevention programs should start during adolescence so as to lay the foundations for a healthy sexual lifestyle.

## Supporting Information

S1 File Ethics Approval. (PDF)
