# Supplementary material for: Determinants of Heterosexual Adolescents Having Sex with Female Sex Workers in Singapore
Source: PLoS One. 2016 Jan 25;11(1):e0147110. doi: 10.1371/journal.pone.0147110 (PMC4726580; doi:10.1371/journal.pone.0147110)
Supplement: S1 File — (PDF) [file pone.0147110.s001.pdf]

National Healthcare Group
Adding years of healthy life

6 Commonwealth Lane
Level 6 GMTI Building
Singapore 149547
Tel: 6496 6600 Fax: 6496 6870
www.nhg.com.sg
RCB No. 200002150H

DSRB Ref: **E/09/073**

20 April 2009

Dr Tan Hiok Hee
Department of Dermatology
National Skin Centre

Dear Dr Tan,

NHG DOMAIN-SPECIFIC REVIEW BOARD (DSRB) APPROVAL

Project Title: A Randomized Controlled Trial of an STI/HIV/AIDS Prevention Intervention for Adolescents Attending A Public STI Clinic in Singapore

We are pleased to inform you that the NHG Domain Specific Review Board has approved the above research project to be conducted in National Skin Centre.

The documents reviewed are:

a) IRB / DSRB Application Form for project titled above: **Version 1**
b) Participant Information Sheet and Consent Form: **Version 2, dated 26 March 2009**
c) Appendix A - Baseline Questionnaire : **Version 1, dated 18 December 2008**
d) Appendix A – Follow-up A (6 months) Questionnaire : **Version 1, dated 18 December 2008**
e) Appendix A - Follow-up B (12 months) Questionnaire: **Version 1, dated 18 December 2008**

The approval period is from **20 April 2009 to 19 April 2010.** The reference number for this study is **DSRB-E/09/073**. Please use this reference number for all future correspondence.

Continued approval is conditional upon your compliance with the following requirements:

1. Only the approved Participant Information Sheet and Consent Form should be used. It must be signed by each subject prior to initiation of any protocol procedures. In addition, each subject should be given a copy of the signed consent form.

2. No deviation from, or changes of the protocol should be implemented without documented approval from the NHG DSRB, except where necessary to eliminate apparent immediate hazard(s) to the study subjects, or when the change(s) involves only logistical or administrative aspects of the trial (e.g. change of monitor or telephone number).

National Healthcare Group

DSRB Ref: **DSRB-E/09/073**

3. Any deviation from, or a change of, the protocol to eliminate an immediate hazard should be promptly reported to the NHG DSRB within seven calendar days.

4. Please submit the following to the NHG DSRB:

   a. All unanticipated problems involving risk to subjects or others, including serious adverse events (SAE) should be reported. In order to assist the DSRB, all reports should be accompanied by the NHG DSRB Unanticipated Problems Involving Risk to Subjects or Others Reporting Form. Please find all forms and guidelines on reporting on the internet at www.b2bresearch.nhg.com.sg.

   b. Report(s) on any new information that may adversely affect the safety of the subject or the conduct of the study.

   c. NHG DSRB Project Status Report Form – this is to be submitted 4 to 6 weeks prior to expiry of the approval period. The study cannot continue beyond **19 April 2010** until approval is renewed by the NHG DSRB.

   d. Study completion – this is to be submitted using the NHG DSRB Project Status Report Form within 4 weeks of study completion or termination.

5. We are happy to inform you that the NHG Research QA Program has been launched in May 2006. The program aims to promote responsible conduct of research in a research culture with high ethical standards, and to identify potential systemic weaknesses and make recommendations for continual improvement. This research project may be randomly selected for completion of self assessment worksheet or for a study review by the QA team. For more information please visit www.b2bresearch.nhg.com.sg.

Yours sincerely,

Dr Ng Swee Cheng
Chairperson
NHG Domain Specific Review Board E

Cc: Director of Research, NSC (via fax only)
c/o Research Department, NSC

Study Reference Number: 2009/00073

Version Number: 5

Please select the appropriate form for submission to the DSRB. Please refer to the explanatory notes below if you need more information.

- ◉ DSRB Application Form 1 - Non Exempt Category
- ○ DSRB Application Form 2 - Exempt Category

Research activities in which the only involvement of human subjects will be in one or more of the following categories may be able to qualify for the Exempt category.

Please click on the DSRB Application Form 2 - Exempt Category option above to view the categories.

DSRB Application Form 1 - Non Exempt Category

Principal Investigators should use Application Form 1 if their research activity does not qualify under the Exempt Category. Application Form 1 should be used for submissions for the Full Board Review and Expedited Review.

DSRB Application Form 2 - Exempt Category

Research activities in which the only involvement of human subjects will be in one or more of the following categories may be able to qualify for the Exempt category.

IMPORTANT: The criteria for the Exempt category do not apply when the research activity:

(i) involves prisoners

(ii) involves children, when the research involves survey or interview procedures or observations of public behavior, except when the investigator(s) do not participate in the activities being observed

(iii) is a US FDA-regulated research activity.

A1 Please enter the full title for this study.

A Randomized Controlled Trial of an STI/HIV/AIDS Prevention Intervention for Adolescents Attending A Public STI Clinic in Singapore

A2 Study Administrators are persons who are responsible for administrative matters related to the Study. They can be the Study Coordinators, Research Nurses or Clinical Research Associates, and need not be part of the Study Team.

While the Principal Investigator remains the primary contact person, the DSRB may contact the Study Administrators for clarification of administrative matters related to the Study.

Study Administrators may also assist the PI in drafting the various online forms and reports, however, only the PI may 'submit' these online forms and reports to the DSRB.

This section is optional but PI's are encouraged to nominate at least one Study Administrator. You may assign Study Administrators for this study below.

No.	Name	Institution	Department	Role	Email
1	Mei Yee Chin	National Skin Centre	Dermatology	Study Administrator	giseleong@ nsc.gov.sg
2	Peiying Lu	National Skin Centre	Administration	Study Administrator	veronlu@ nsc.gov.sg
3	Junice Yi Siu Ng	NUS - Saw Swee Hock School of Public Health	NUS - Saw Swee Hock School of Public Health	Study Administrator	junice@ nus.edu.sg

B1 Study Sites & Study Team Members

All investigators who have a responsibility for the consent process and/or direct data collection for this study should be listed below.

Study Team Members with registered user account with us will be notified of their participation in this study when the Application is submitted.

For a Multi-centre study, please appoint a Site PI for each site (Mandatory).

The Principal Investigator will be the Site PI for their own Institution, and will also be the primary contact person for the DSRB.

(i) 'Overall Principal Investigator': PRIYA SEN

(ii) Study Sites under the oversight of NHG DSRB Click here for help

No.	Study Site	Name	Study Role	Institution	Department	Min Training
1	National Skin Centre	Dr PRIYA SEN	PI	National Skin Centre	Dermatology	Completed
2	National Skin Centre	Prof roy chan	Co-Investigat or	National Skin Centre	Dermatology	Completed
3	National Skin Centre	Dr Martin Chi o	Collaborator	National Skin Centre	Dermatology	Completed
4	National Skin Centre	Dr Hiok Hee T an	Collaborator	National Skin Centre	Dermatology	Completed
5	NUS - Saw Sw ee Hock Scho ol of Public H ealth	A/Prof Mee Li an Wong	Site PI	NUS - Saw Sw ee Hock Scho ol of Public H ealth	NUS - Saw Sw ee Hock Scho ol of Public H ealth	Not complete d
6	NUS - Saw Sw ee Hock Scho ol of Public H ealth	Prof David Ko h	Co-Investigat or	NUS - Saw Sw ee Hock Scho ol of Public H ealth	NUS - Saw Sw ee Hock Scho ol of Public H ealth	Not complete d
7	NUS - Saw Sw ee Hock Scho ol of Public H ealth	Dr Judy Sng	Co-Investigat or	NUS - Saw Sw ee Hock Scho ol of Public H ealth	NUS - Saw Sw ee Hock Scho ol of Public H ealth	Completed

(iii) Other external Study Sites under the supervision of the 'Overall Principal Investigator' (eg. Nursing Home, Community Hospitals, Community Centres etc)

B2 External Study Site (for Institutions NOT under the oversight of NHG DSRB)

(i) Are there any other independent study sites by another PI which are conducting the same study?

○ Yes

◉ No

B3 Research Specialty

Please select the Primary Specialty, and then choose the relevant Sub specialty that has been matched according to the Primary Specialty selected. If the Primary Specialty and/or Sub specialty cannot be found from the list, please choose 'Others' and specify.

No.	Primary Specialty	Primary Sub Specialty
1	Dermatology	Cutaneous Infections

Please indicate/add Secondary Specialties.

No.	Primary Specialty
There is nothing.	

B4

i. Which Domain Specific Review Board (DSRB) is this application being submitted to? DSRB Domain E

ii. Has the study been submitted to another IRB?

◉ No
○ Yes

iii. Has the application been previously rejected by any IRB? (Including NHG-DSRB)

◉ No
○ Yes

The Conflict of Interest Declaration section must be completed by the PI on behalf of the Study Team if any member of the Study Team has any potential conflicting interest while conducting the research. This Declaration also includes any Conflict of Interests of their immediate family members (includes spouse and each dependent child). Any such member(s) must complete and submit their Declarations when this application is submitted. The PI is responsible for checking and ensuring that accurate information is submitted to the DSRB.

Conflicting Interest - A conflicting interest can be broadly defined to refer to any interest of the investigator that competes with the investigator's obligation to protect the rights and welfare of research subjects.

Financial Interest - Significant Financial Interest means anything of monetary value, including but not limited to, salary or payments for services (e.g. consulting fees or honoraria); equity interests (e.g. stocks, stock options or other ownership interests); intellectual property rights (e.g. patents, copyrights and royalties from such rights), and board or executive relationships.

The Conflict of Interest Declaration Section must be submitted to the DSRB via study amendments if any of the circumstances relevant described herein change during the conduct of the research.

Dr PRIYA SEN (Principal Investigator)
- ○ Yes
- ◉ No

Prof roy chan (Co-Investigator)
- ○ Yes
- ◉ No

Dr Martin Chio (Collaborator)
- ○ Yes
- ◉ No

Dr Hiok Hee Tan (Collaborator)
- ○ Yes
- ◉ No

A/Prof Mee Lian Wong (Site Principal Investigator)
- ○ Yes
- ◉ No

Prof David Koh (Principal Investigator)
- ○ Yes
- ◉ No

Dr Judy Sng (Co-Investigator)
- ○ Yes
- ◉ No

This is a smart form. The choice you make here will determine which sections of the application form will appear.

Clinical Trials

Choose this if your research involves:

(1) Administering a drug, device, or biologic as part of the research intervention, or

(2) Performing surgical procedures as part of research intervention

Questionnaire/ Survey/ Interviews

Choose this if your research involves:

(1) Administering questionnaires/surveys/interviews. This type of research may also include a medical records review component.

Medical Records Review

Choose this if your research involves:

(1) Collection of data for a specific research project by review of medical records including results of routine diagnostic tests performed for standard clinical purposes

(2) Prospective and/or retrospective data collection

Clinical Research

Choose this if your research involves:

(1) Collection of blood by venepuncture, finger stick, etc or

(2) Prospective collection of biological specimen by invasive or non invasive means including biopsies, FNAC's, fundoscopy etc or

(3) Collection of data through research procedures such as X rays, MRI, ultrasound, ECG, EEG, etc or

(4) Any other research categories that are not listed in the options above.

D1 Please select one category that best describes your research activities.

○ Clinical Trials (which includes Drug, Device and Surgical-Procedure Trials)
◉ Questionnaire/ Survey/ Interviews
○ Medical Records Review
○ Clinical Research

Note: Clinical Trial Certificate from Health Sciences Authority might be required if you are testing the safety and efficacy of the medicinal product. You should check with HSA if you are unsure.

D2 Is this a US FDA IND/IDE study or data is intended to be reported to FDA in support of a IND/ IDE application?

○ Yes
◉ No

Note: US FDA-regulated (IND) research activities cannot qualify for Exemption from DSRB Review and Waiver of Informed Consent. The application must be submitted using the DSRB Application Form 1 - Non Exempt Category.

D3 Is this study subjected to any of the following regulations:

◉ No
○ Yes

☐ US Code of Federal Regulations 45 CFR 46
☐ US Code of Federal Regulations 21 CFR 50
☐ US Code of Federal Regulations 21 CFR 56

☐ US Code of Federal Regulations 21 CFR 312
☐ US Code of Federal Regulations 21 CFR 812
☐ Others

E1 Who will be responsible for the payment and compensation of injury or illness arising from participation of subjects in the study?

The PI should ensure that insurance coverage is available to provide payment and compensation to research subjects for injury or illness arising from their participation in the study.

(Note: For investigator-initiated studies - Contact your OBR/CRU for more information on available NHG Clinical Trial Compensation Insurance Scheme.

For Sponsored Studies - Sponsors should be primarily responsible for ensuring that subjects receive payment and compensation in the event of injury or illness as a result of their participation in a research study.)

National Skin Centre

E2 Please give information regarding the study's Funding source or Sponsor information.

○ No funding is required for this study to be carried out
○ Pharmaceutical / Industry Sponsored
◉ Grant

i. Name of Grant Agency and Grant Name Others

Please specify: National Medical Research Council

ii. Grant amount applied for 582400.0

iii. Date of Grant application deadline 01-Aug-2008

iv. Has the Grant application been approved?

◉ Yes. Grant application successful.

Date of Grant Approval: 01-Nov-2008

Date of Grant Expiry: 31-Dec-2014

Amount of Grant awarded: 582400.0

Please attach the approved grant proposal and all relevant documents approved by the grant body(e.g. study protocol,consent form ect)

○ No. Grant application is pending approval.

E3 Who will be responsible for research-related costs? For sponsored studies, please list the costs that will be borne by the sponsor. You may wish to attach the Financial Agreement / Clinical Trial Assurance if it is available. * Click here for help

NMRC

F1 Please provide an abstract of your proposed research (Up to 300 words).

Your abstract must contain:

Aims

Methodology

Importance of proposed research to science or medicine

Potential benefits & risks

AIMS:This study tests the hypothesis that a comprehensive HIV/AIDS and sexually transmitted infections (STI) risk reduction program that addresses social, behavioral and environmental influences on risky sexual behaviors among sexually experienced adolescents will achieve a sustained reduction in HIV/STI risk behaviors and HIV/STI morbiditySpecifically, this study aims to:1. Assess the effectiveness of an STI/HIV/AIDs prevention intervention in enhancing life skills and promoting secondary sexual abstinence among sexually experienced adolescents attending the public STI clinic in Singapore.2. Assess the effectiveness of an STI/HIV/AIDs prevention intervention in reducing risky sexual behaviors such as unprotected sex among these adolescents 3. Assess the impact of the intervention in reducing incident sexually transmitted infections among them 4. Identify program and participant variables associated with effective changes in behavior. METHODOLOGY:A randomized controlled trial will be conducted in which adolescent patients will be randomly assigned either to the new STI prevention program or the existing program conducted by DSC (control group). The existing program, which has not been evaluated, consists of 2 individual counseling sessions - the first session at first attendance and the second session 2 weeks later following confirmation of diagnosis by laboratory tests. Only those tested positive attends a second counselling session. Those who are tested negative are informed by handphone and are not required to attend the second session. Both groups will be followed up for 12 months.For the intervention group, a comprehensive evidence-based program will be developed applying principles from Green's PRECEDE PROCEED framework, Social Cognitive Theory, Theory of Gender and Power as well as important findings from our case control study (which has been accepted for publication in Pediatrics, a top tier journal) on factors associated with sexual initiation, condom use and other risky sexual behaviour among these adolescents. Strategies used in the AIDS Risk Reduction Education and Skills Training Program and from other tried and tested programs will be adapted to be socioculturally appropriate to our three main ethnic groups. Input will be obtained from social workers, psychologists, youth workers and religious leaders who have experience working with youths. The intervention aims to promote sexual abstinence and/or safer sex through providing information on STI/HIV risks and prevention, communication and assertiveness training skills, life skills such as goal setting, problem solving as well as supporting parents and teens to facilitate behaviour change. These will be delivered via a combination of activities ranging from online video clips,skills development sessions, new media such as email, blogs, interactive elearning and websites providing information to individual counseling and group discussions. Proactive telephone or electronic support rather than reactive support will be employed to follow-up adolescents who defaulted on sessions. All adolescents enrolled in the intervention will be given a password to log on to an online portal/website with information on STI prevention and lifeskills designed specifically for them. For the control group, as it is unethical to withhold information which will benefit the participants, those found to be re-infected with STIs at the follow-up will be given individual counselling. In summary, the intervention differs from the routine program in having (i) follow up for all sexually active teens, including those testing negative, (ii) higher frequency of sessions (6 versus 1-2 for the existing programme, (iii) referral to trained counsellors identified by DSC for continuing social and emotional support (iv) skills building activities and lifeskills development which range from communication skills and assertiveness training delivered through video demonstrations, group sessions and proactive telephone and electronic support .INTROIMPORTANCE:Adolescents are experiencing the most rapid increase in HIV worldwide, with about half of new HIV infections occurring among young people aged 15-24. This multidisciplinary study involving experienced researchers from the fields of public health, clinical medicine (STIs) and occupational health will provide pertinent data for planning effective STI/HIV prevention interventions for adolescents not only in Singapore but also in the region, where HIV is rising markedly.POTENTIAL BENEFITS:All study participants who have acquired sexually transmitted infections will be treated appropriately at DSC and counselled on abstinence and on how to protect themselves from being reinfected with STIs, regardless of

whether they are assigned to the intervention or control group. All participants in the intervention group will receive additional counselling sessions targeted at reducing high risk behaviour.POTENTIAL RISKS:There is a very small risk that obtaining blood may cause pain, bleeding, bruising, or swelling at the site of the needle stick, occasionally associated with fainting and rarely infection. However, this is less likely as only the trained staff at the DSC (Department of STI Control) or National Skin Centre (NSC) laboratory will be performing the blood taking on the study participants. DSC or NSC laboratory has very well trained staff and the lab has been accredidated by the Singapore Laboratory Accredidation Scheme.The risk of breach of confidentiality will be minimised by having only codes on the questionnaire. Researchers will not have the names or identifiers of the participants. Only the DSC or NSC staff in charge of patient care of the adolescents will have access to the identifiers, as they need to ensure that laboratory reports are correctly matched so that correct treatment is given to those who need it. However, this staff will not have access to the questionnaires or research database. Hence the data have been delinked with no one person having all the information.

F2 What are the Specific Aims of this study?

This study tests the hypothesis that a comprehensive HIV/AIDS and sexually transmitted infections (STI) risk reduction program that addresses social, behavioral and environmental influences on risky sexual behaviors among sexually experienced adolescents will achieve a sustained reduction in HIV/STI risk behaviors and HIV/STI morbiditySpecifically, this study aims to:1. Assess the effectiveness of an STI/HIV/AIDs prevention intervention in enhancing life skills and promoting secondary sexual abstinence among sexually experienced adolescents attending the public STI clinic in Singapore.2. Assess the effectiveness of an STI/HIV/AIDs prevention intervention in reducing risky sexual behaviors such as unprotected sex among these adolescents 3. Assess the impact of the intervention in reducing incident sexually transmitted infections among them 4. Identify program and participant variables associated with effective changes in behavior.

F3 What is the Hypothesis of this study?

A comprehensive HIV/AIDS and sexually transmitted infections (STI) risk reduction program that addresses social, behavioral and environmental influences on risky sexual behaviors among sexually experienced adolescents will achieve a sustained reduction in HIV/STI risk behaviors and HIV/STI morbid

F4 Please briefly describe the background to the current study proposal. Critically evaluate the existing knowledge and specifically identify the gaps that the proposed study is intended to fill.

2.1 HIV/AIDS/STIs and sexual behaviour among adolescents In Singapore, the incidence of STIs among adolescents aged 10 to 19 years has increased by more than 3-fold from 250 cases (3.45% of all cases) in 2002 to 820 cases (7.06% of infections) in 2007. This rate of increase is the highest compared to other age groups and is expected to continue. A preliminary analysis of an ongoing study by the principal investigator and team on 500 unmarried but sexually experienced adolescents aged 14 to 19 years attending DSC found a high prevalence of STIs (60%), young age of sexual initiation (lowest age:9 years), high number of sex partners (median:4 range:1-25) and high percentage of unprotected sex (90%). The age of sexual debut has fallen markedly from 16 years in 1992 to 9 years in 2006. The adolescents also markedly underestimated their risk of STI. Almost half of those who thought that they had no chance at all of having STI before testing were in fact confirmed to have STI following testing. A high proportion of them (49%) continued to engage in unprotected sex and three quarters of them delayed seeking treatment for more than a week after experiencing symptoms of STIs. These findings clearly call for an urgent need to develop interventions for adolescents to prevent risky sexual behaviors among them. A recent review conducted on abstinence-plus programs (promoting sexual abstinence as the best means of preventing HIV, but also encouraging condom use and other safer-sex practices such as reduction of partners) found that these interventions appear to reduce short-term and long-term HIV risk behavior among youth in high-income countries. As all the trials on the abstinence-plus programs were conducted in North America, it is not known whether such interventions will work in Singapore or Asian populations, given their contextual differences in socio-cultural, structural and environmental influences. 2.2 Gaps in research Presently, there is a lack of HIV/STI prevention programs targeting high risk groups of sexually experienced adolescents in Asia. While it is important to implement programs for adolescents in the general population, specific strategies targeting high-risk sexually experienced adolescents who may be infected with STIs deserve priority because they serve as a core group for transmission of STIs to other adolescents in the community. In addition, none of the published studies on STI/HIV prevention intervention programs for adolescents in Asia used biological end points such as STIs or HIV to evaluate program effectiveness. To date, no study has been conducted to assess the efficacy of interventions to reduce risky sexual behaviors among adolescents in Singapore. 2.3 Justification for conducting

the study on adolescents attending the public STI clinic Our targeted approach on adolescents attending the Department of STI Control clinic (DSC) will make the greatest impact in reducing new cases of STIs among them in the long term because this is a high risk core group for transmitting STIs to the general population.

F5 Please provide a list of relevant references.

1. Joint United Nations Program on HIV/AIDS (UNAIDS) and World Health Organization (WHO) AIDS epidemic update-December 2006.2. Chan RKW, Tan HH. Sexually transmitted Infections in Singapore Teenagers. Ann Acad Med Singapore 2003; 32:25-8.3. Department of STI Control. Annual Report 2007, Singapore: Department of STI Control, 2008.4. Underhill K, Montgomery P, Operario D. Sexual abstinence only programmes to prevent HIV infection in high income countries:systematic review. BMJ 2007; Aug 4;335(7613):248-250.5. Ralph J. DiClemente, Gina M. Wingood, Kathy F. Harrington, Delia L. Lang, Susan L. Davies, Edward W. Hook et al Efficacy of an HIV Prevention Intervention for African American Adolescent Girls: A Randomized Controlled Trial. JAMA. 2004;292:171-179.

F6 Please submit a copy of at least two relevant papers.

DSRB2_BMJ syst rev Abstinence only programmes.pdf

JAMA HIV prevention RCT.pdf

F7 Please state concisely the importance of the research described in this application by relating the specific aims to the long term objectives.

Adolescents are experiencing the most rapid increase in HIV worldwide, with about half of new HIV infections occurring among young people aged 15-24. This multidisciplinary study involving experienced researchers from the fields of public health, clinical medicine (STIs) and occupational health will provide pertinent data for planning effective STI/HIV prevention interventions for adolescents not only in Singapore but also in the region, where HIV is rising markedly.

F8 Discuss in detail the experimental design and procedures to be used to accomplish the specific aims of the study. (If this study involves a retrospective medical record review, please specify the period of data collection.) Note: W.e.f. 1 July 2014, all research studies submitted from National University Hospital (NUH), involving the use of radioactive materials and/or radiation-emitting equipment will need to obtain approval from the NUH Radiation Safety Committee (RSC) prior to the commencement of the study. For more information and to receive a copy of the 'Guidelines to undertake Research which involves the use of Ionizing and/or Non-Ionizing Radiation', please contact the NUH Radiation Safety Officer (michael_tong@nuhs.edu.sg) or the NUHS Research Office (clinical_research@nuhs.edu.sg).

A randomized controlled trial will be conducted in which adolescent patients will be randomly assigned either to the new STI prevention program or the existing program conducted by DSC (control group). The existing program, which has not been evaluated, consists of 2 individual counseling sessions - the first session at first attendance and the second session 2 weeks later following confirmation of diagnosis by laboratory tests. Only those tested positive attends a second counselling session. Those who are tested negative are informed by handphone and are not required to attend the second session. Both groups will be followed up for 12 months.For the intervention group, a comprehensive evidence-based program will be developed applying principles from Green's PRECEDE PROCEED framework, Social Cognitive Theory, Theory of Gender and Power as well as important findings from our case control study (which has been accepted for publication in Pediatrics, a top tier journal) on factors associated with sexual initiation, condom use and other risky sexual behaviour among these adolescents. Strategies used in the AIDS Risk Reduction Education and Skills Training Program and from other tried and tested programs will be adapted to be socioculturally appropriate to our three main ethnic groups. Input will be obtained from social workers, psychologists, youth workers and religious leaders who have experience working with youths. The intervention aims to promote sexual abstinence and/or safer sex through providing information on STI/HIV risks and prevention, communication and assertiveness training skills, life skills such as goal setting, problem solving as well as supporting parents and teens to facilitate behaviour change. These will be delivered via a combination of activities ranging from online video clips,skills development sessions, new media such as email, blogs, interactive elearning and websites providing information to individual counseling and group discussions. Proactive telephone or electronic support rather than reactive support will be employed to follow-up adolescents who defaulted on

sessions. All adolescents enrolled in the intervention will be given a password to log on to an online portal/website with information on STI prevention and lifeskills designed specifically for them. For the control group, as it is unethical to withhold information which will benefit the participants, those found to be re-infected with STIs at the follow-up will be given individual counselling. In summary, the intervention differs from the routine program in having (i) follow up for all sexually active teens, including those testing negative, (ii) higher frequency of sessions (6 versus 1-2 for the existing programme, (iii) referral to trained counsellors identified by DSC for continuing social and emotional support (iv) skills building activities and lifeskills development which range from communication skills and assertiveness training delivered through video demonstrations, group sessions and proactive telephone and electronic support .

F9 Please provide details on sample size and power calculation and the means by which data will be analyzed and interpreted (If applicable). * Click here for help

Already submitted and covered

see F3

F11 List all activities that are performed for routine diagnostic or standard medical treatment as part of the subject's standard care. All research-related activities should not be stated in this section.

n.a.

F12 Please describe the subject's visits (frequency and procedures involved). For studies with multiple visits, please attach study schedule. (If applicable)

Study schedule as in protocol

F13 Discuss the potential difficulties and limitations of the proposed procedures and alternative approaches to achieve the aims.

There may be social desirability bias from self-reported condom use and sexual behavior, which will be minimised by assuring confidentiality and stressing that the study aims to help protect them from STIs/HIV/AIDS. Interviewers, not involved in conducting the program sessions, will be trained to conduct the survey and will be blinded to participants' condition assignments. Sensitive questions on sexual behavior will be self-administered by participants. Some factors associated with risky sexual behaviors such as dysfunctional families and delinquency are complex and may not be modifiable; hence attrition may be high among this group. We will attempt to reduce attrition by sending reminder calls to their individual handphones; providing transport and food allowances of S$30 per person per visit and decentralizing follow-up sessions to increase access. A comparison will be made between those who drop out and those who participate in the study.To protect the confidentiality of the patient, we will not be sending reminder letters as other family members in the household may open the letter. As all adolescents recruited in our research project will have a password to access an online portal/website designed specifically for them, we'll post a simple reminder to come to the DSC for follow-up with a simple non-specific message "Please remember to attend your lifeskills class at our "Yahoo project on Living well."The name of the clinic or the programme will not to be specified in the message.While recruiting participants to our research project, we found that some participants (approximately 40%) have difficulty in attending follow-up sessions at DSC clinic during office hours due to work, study, inconvenience or embarrassment of coming to the clinic. Our participants also did not like to go to the National Skin Centre where laboratory tests can be done because they did not like to attend a clinic setting. Given the above reasons, we conduct follow-up sessions at other venues preferred by the participants to increase follow-up rates. Follow-up counseling sessions will hence be conducted at Saw Swee Hock School of Public Health, National University of Singapore, Health Promotion Board, the SCAPE building or other venues of the participants' choice eg a quiet corner in a restaurant, void deck of an HDB estate. Outreach follow-up individual and group counseling for these participants will be conducted during daylight hours (from 9 am to 6.30 pm) on weekdays and weekends to increase access and acceptability for the patients. Urine samples will be taken without blood tests outside DSC for the abovementioned group of study participants at the above-mentioned venues. Urine test kits will be provided to our trained counselors. Participants are required to fill up half or three quarters of the plastic bottle with urine. This is a safe non-invasive test and the procedure can be easily administered by the participants. Participants who attend DSC clinic collect their urine using the same procedure as well. The trained counselors will then transfer the urine to a test tube using a sterile

syringe in the urine test kit pack as listed in Appendix 1.Outreach services conducted at National University of Singapore (NUS), Health Promotion Board (HPB)/SCAPE building will be held in quite private conducive rooms. For other venues of the participants' choice eg a quiet corner in a restaurant, void deck of an HDB estate, there are no rooms available. We will take special care to conduct the session in a place or corner that is discreet and private to ensure the confidentiality and comfort of the respondents, e.g. void decks of HDB estates and parks.

F14 What are the Potential Risks to Subjects?

There is a very small risk that obtaining blood may cause pain, bleeding, bruising, or swelling at the site of the needle stick, occasionally associated with fainting and rarely infection. However, this is less likely as only the trained staff at the DSC (Department of STI Control) or National Skin Centre (NSC) laboratory will be performing the blood taking on the study participants. DSC or NSC laboratory has very well trained staff and the lab has been accredidated by the Singapore Laboratory Accredidation Scheme.The risk of breach of confidentiality will be minimised by having only codes on the questionnaire. Researchers will not have the names or identifiers of the participants. Only the DSC or NSC staff in charge of patient care of the adolescents will have access to the identifiers, as they need to ensure that laboratory reports are correctly matched so that correct treatment is given to those who need it. However, this staff will not have access to the questionnaires or research database. Hence the data have been delinked with no one person having all the information.

F15 What are the Potential Benefits (direct as well as indirect) to subjects? Indirect benefit may refer to the medical knowledge gained in the future, from the research.

All study participants who have acquired sexually transmitted infections will be treated appropriately at DSC and counselled on abstinence and on how to protect themselves from being reinfected with STIs, regardless of whether they are assigned to the intervention or control group. All participants in the intervention group will receive additional counselling sessions targeted at reducing high risk behaviour.

F16 Preliminary Studies / Progress Reports. Please provide an account of the Principal Investigator's preliminary studies (if any) pertinent to this application.

This proposed study is an extension of an earlier NMRC-funded case control study (March 2006-February 2008) by the PIs and their multidisciplinary research team (involving an occupational medicine specialist, STI consultant physician and psychologist) to assess factors associated with STIs and high risk sexual behaviors among adolescents attending the public Department of STI Control clinic (DSC) in Singapore. The alarming findings on their young age of sexual initiation (youngest: 9 years), multiple partners(median: 5), high prevalence of unprotected sex (90%), and sexually transmitted infections(60 per 100 persons) coupled with the lack of health care services targeted specifically for adolescents have prompted the PI and team to plan this study on the development and evaluation of interventions for high risk adolescents.

F17 What is the estimated timeline for this study? Click here for help

Estimated Start Date: 15-Apr-2009

Estimated End Date: 31-Dec-2014

F18 Does this study have a Study Protocol? Note: For Clinical Trials, investigators are required to submit a Study Protocol for review.

○ Yes
◉ No

F19 The PI is responsible for ensuring that all Study Subjects give informed consent before enrolling into the study.

Please select all the applicable consent scenarios.

◉ Informed Consent will be taken for all study subjects.
○ Waiver of Informed Consent is requested for all study subjects.

○ A combination of both Informed Consent and Waiver of Consent is required for different study populations.

H1 How will potential subjects be identified? (Please tick all the applicable boxes)

☑ Referral by attending healthcare professional
☐ Patients of study team
☐ Databases
☐ Other methods of subject identification

H2 Who will make the first contact with subject (Enter NA if not applicable)? * Click here for help

Staff nurse and trained interviewer. In view of the slow recruitment rate of 10 participants per month, we have designed a new poster to be distributed to healthcare professionals and school counselors. The patients will be referred to the DSC clinic. In addition, we are also writing to the relevant agencies, such as family service centres to refer cases to DSC (see attached letter and list of contacts).

Patients attending DSC will be referred to the interviewer by the staff nurse who screens the patients before their consultation with the doctor. All adolescents who fit into the inclusion criteria will be referred to the interviewer.

◉ Yes

Please tick all the applicable types of advertising / recruitment materials that will be used in this study.

☑ (i) Posters

Please state the locations(s) where the posters will be placed (e.g. In the hospital lift, in the general waiting area in clinic), and attach a copy of the poster.

The materials will be be distributed to healthcare professionals such as medical social workers, doctors, nurses in hospital adolescent units/GPs/polyclinics.

ABI Flyer_version 2 dated 28Nov2012.jpg

☐ (ii) Brochures
☐ (iii) Advertisements in Newspapers / Magazines / Publications
☐ (iv) Advertisements on Radio / TV.
☐ (v) 'Letter of Invitation' to potential research participants. 'Letter of Invitation' refers to email, letters or any form of documents used as part of the recruitment strategy, with the intention of inviting the research participants to participate in the study. Please attach a copy of the Letter of Invitation for DSRB Approval before use.
☐ (vi) Letter to Doctors requesting for referrals.
☐ (vii) Other types of materials will be used.

○ No

H5 Will any other recruitment strategies be used? (Eg. Talks in public places, societies etc.)

○ Yes
◉ No

H6 What is the Recruitment Period (if applicable)? Please provide us with the approximate recruitment period. Click here for help

Start Date: 01-Feb-2009

End Date: 28-Feb-2011

H7 Please indicate the length of time of the subject's direct involvement in the study. E.g. For clinical visits, examinations etc. (If applicable)

Duration of subject involvement in research: 12 months from time of recruitment

I1 Please state the target number of research subjects to be recruited for each study site in Singapore. If the exact numbers are not available, please give an approximate number range in the Recruitment Target Minimum and Maximum columns.*

Please note that recruiting subjects beyond the Max. No. without DSRB's approval would constitute a Non-Compliance. If you intend to recruit beyond the Max. No., please submit a study amendment to increase the recruitment target.

For the distribution of Males, Females and Children to be recruited into the study, please use the Recruitment Target Max. No. to provide an approximate distribution ratio.

(Go back to Section B1 to add additional study site)

No.	Study Site	Recruitment Target Min	Recruitment Target Max	Males	Females	Children
1	NUS - Saw Swee Hock School of Public Health	800	800	400	400	0
2	National Skin Centre	0	0	0	0	0

I2 Is this study part of an international study?

○ Yes
◉ No

K1 Please list the inclusion criteria for research subjects in this study. Note: For global studies, please modify the criteria according to local regulations (e.g. persons below the age of 21 are considered minors in Singapore and would require parental consent prior to participation).

Singaporean citizens or permanent Singaporean residents aged between 16 and 19 years who are sexually active, unmarried, attending the clinic for the first time and provide written consent.

K2 Please list the exclusion criteria for research subjects in this study. Please state clearly, if pregnant women will be excluded from the study.

Foreigners, cases of rape and male/female adolescents engaging in sex with the same gender will be excluded. Adolescents who are bisexual, ie. engaging in sex with both same and opposite gender will be excluded.

K3 Please state the age group of the research subjects.

Lower Age limit 16 Lower Age option years

Upper Age limit 19 Upper Age option years

K4 Are there any recruitment restrictions based on the gender of the research subjects?

○ Yes
◉ No

K5 Are there any recruitment restrictions based on the race of the research subjects (e.g. only chinese subjects will be included in this study)? If 'Yes', please provide a rationale for this race restriction.

○ Yes
◉ No

K6 Do the potential research subjects have a dependent relationship with the study team (E.g. doctor-patient, employee-employer, head-subordinate, student-teacher, departmental staff relationship)? Note: If you have selected that subjects are 'Patients of study team' in Section H1, then the answer should be 'Yes'. * Click here for help

○ Yes
◉ No

◉ Yes

Please select all the applicable categories.

- ☐ **Pregnant Women, Foetuses and Neonates**
- ☑ **Children (persons who are less than 21 years of age)**
- ☐ **Prisoners**
- ☐ **Cognitively Impaired persons**
- ☐ **Others (E.g. mentally disabled persons, or economically or educationally disadvantaged persons.)**

○ No

K8 Does the study involve any of the following?

- ☐ Inpatients
- ☑ Outpatients
- ☐ Healthy Volunteers
- ☐ Not applicable

M Research involving Children (Persons under the age of 21 years) - Please provide protocol specific information explaining how your proposed research project meets the following criteria.

M1 Describe if appropriate studies have been conducted on animals and adults first, and data is available to assess risks to children participating in the research.

Not applicable as this is a behavioural intervention study and not a clinical/drug trial.

M2 Please justify the need to involve children. Can the research question be answered through alternative means (e.g. involving adults only)?

This study aims to address the problem of the alarmingly rapid increase of sexually transmitted infections among adolescents aged 16 to 19. Therefore the involvement of participants of this age group is central to the research question and it cannot be answered through alternative means.

M3 Describe how the relation of potential benefits to risks is at least as favourable as that presented by alternative approaches.

The potential benefits are that high-risk sexual behaviour and incidence of sexually transmitted infections (as well as HIV, unwanted pregnancy) will be reduced. There are no risks to participants.

M4 Describe any additional safeguards that will be provided to protect the rights, safety and welfare of these vulnerable subjects.

The Fraser guidelines (Appendix A) on testing and treatment of under-aged persons will be adhered to and patient anonymity and confidentiality will be maintained. According to the guidelines, the age of consent (or the age above which consent from the parent/guardian is not necessary) is 16 years or older. For patients aged 16 to 19, informed consent will be taken from each participant and he or she will be given an information sheet to read before deciding whether to participate. Further explanations will also be given to the potential participant by the interviewer.

M5 What are the provisions for obtaining the child's assent and parental permission? (Check all that apply) * Click here for help

○ Assent will be obtained from all children above 6 years old and Parental Permission will be obtained.
○ Assent will not be obtained from the children. Only Parental Permission will be obtained.
◉ Parental Permission will not be obtained from the parents. Only assent will be obtained.

Please justify:

The Fraser guidelines (Appendix A) on testing and treatment of under-aged persons will be adhered to and patient anonymity and confidentiality will be maintained. According to the guidelines, the age of consent (or the age above which consent from the parent/guardian is not necessary) is 16 years or older. For patients aged 16 to 19, informed consent will be taken from each participant and he or she will be given an information sheet to read before deciding whether to participate. Further explanations will also be given to the potential participant by the interviewer.

○ Neither the children's Assent or Parental Permission will be obtained.

P YES. Informed consent will be obtained from potential Research Participants before enrollment into the study.

The PI is responsible for ensuring that all Research Participants give informed consent before enrolling into the study. Please describe the consent process below.

P1 Describe when the consent process will take place with the potential subject. Note: Subjects should be approached prior to the initiation of any study procedures and should not be approached in a situation where they may feel compromised (e.g. while in labour, just prior to a surgical procedure or under sedation).

Consent will be obtained when a patient attending the DSC clinic fits the inclusion criteria and is selected for the study. Prior to obtaining consent, the interviewer will give the Participant Information Sheet to the patient. The interviewer will then explain the study, give time for the patient to read the information sheet and answer any questions he or she may have. Consent will be then be taken. Generally, signed consent forms are given to the participants. However, given the nature of our study, this is not advisable as these signed consent forms bear the names, identity numbers and indicate their visits to DSC. As participants recruited are between the ages of 16-19 years, they are unlikely to bring any documents from DSC clinic home as they may be afraid that their family will find out. There is a possibility that they may discard the signed consent forms with their names in rubbish bins in public places. Hence, we suggest that signed consent forms and information sheets should not be given to participants. Instead, the consent forms will be kept in a secure place in DSC, and the information contained in the PIS will be made accessible to the teens via a website. It is therefore not neccesary to give any consent form (blank or otherwise) to the particpant.

P2 Where will the consent process take place with the potential subject (e.g. in room ward, outpatient clinic etc.)?* Please justify why the place chosen for the consent process is suitable. * Click here for help

Consent will be taken in a consultation room or a private corner of the clinic.

P3 Specify who be involved in taking Informed Consent from the potential subjects (e.g. PI, co-investigators etc.) Note: Only study team members or research assistants who have been delegated by the PI can obtain consent from the subjects. This should be documented in the Study Responsibility Log. It is the responsibility of the PI to ensure that the study staff who are delegated to obtain consent have received proper training (e.g. CITI, SGGCP, PCR course).

A trained counsellor who has a CITI certificate and has experience working in health care and /or with adolescents in DSC will conduct the consent process. As this is an RCT for a behavioural intervention and not a drug trial, it is not necessary for a doctor to be present to explain drug adverse effects and such. It is also not feasible for the investigators to take consent as the investigators are not treating patients at DSC all the time.

See O1

P5 Does your study involve potential vulnerable subjects whereby obtaining informed consent form from the subject is not possible and informed consent is required from a Legally Acceptable Representative (LAR)?

- ◉ No
- ○ Yes

To protect the privacy interests of Study Subjects, only the first section of the questionnaire on socio-demographics will be interviewer-administered. The second section of the questionnaire (also translated into Malay and Chinese), on sexual behaviour will be self-administered. In addition, sessions held in the clinic (interviews, counselling, video screening) will be conducted in private in a consultation room. We aim to have at least 3 of the 6 sessions for the intervention group delivered via the Internet or phone.

P7 Besides the Informed Consent Form, will any other materials or documents be used to explain the study to potential Research Participants? (eg. scripts, handouts, brochures, videos, logs, etc).

- ◉ No
- ○ Yes

- ○ No

◉ Yes

Please provide details of the gifts and payment (including the amount paid).

Participants will be reimbursed S$30 for each follow-up visit to cover meals and transport expenses. For participants who choose outreach services, they will be reimbursed S$20 for each follow-up visit to cover meals and transport costs. The balance will be used to offset the transportation cost for our counselors who conduct the outreach services.We have also designed two new certificates of participation as incentives to increase follow-up rates and to acknowledge the participants' contribution to the project. Participants will receive a certificate of merit if they 'attend' all 4 counseling sessions (including the session by online counselors) and participate actively in the talks, demonstrations and video clip sessions on youth health promotion and life skills development up to a period of 6 months. Participants will receive a certificate of distinction if they 'attend' all 6 counseling sessions (including the session by online counselors) and participate actively in the talks, demonstrations and video clip sessions on youth health promotion and life skills development up to a period of 12 months. (Appendices 5 and 6)

P9 Will consent be documented in the form of a written and signed Informed Consent Form?

◉ Yes, all Research Participants will be given a copy of the Informed Consent Form.

Please attach a copy of the Informed Consent Form.

Particpants Information Sheet_09 May 2012_Tracked.doc

Particpants Information Sheet_5Sep2012_Clean.doc

Informed consent form_version 6 dated 28Nov2012.doc

○ No, Consent will not be documented. (E.g. verbal consent).

P10 Consent Language

(i) Will the study enroll non English speaking subjects?

◉ No
○ Yes

P11 Will the study be recruiting subjects under emergency situations, when prior consent of the subject is not possible, and the consent of the subject's legally acceptable representative, if present, should be requested?

○ Yes
◉ No

P12 Do you have any additional comments regarding the Informed Consent process?

◉ No
○ Yes

R In general, to protect the Study Subject's confidentiality, research data should be coded, and the links between the Subject's identifiers and the codes should be stored separately from the research data.

R1 Will coded / anonymous research data be sent to the study sponsor (e.g. pharmaceutical-sponsored studies)?

◉ No, the study team would store all research data within the institution

i. Please state where the research data (soft copy and/or hardcopy) will be stored and indicate if the location storage is secured (i.e Password Protected PC or Laptop, data stored in physical location with lock and key access.)

Research data will be stored and locked on a stand-alone PC in A/Prof Wong Mee Lian's office in the Department of Community, Occupational and Family Medicine, NUS.The signed Indiviudal Consent Documents (ICFs) will be stored at DSC in a secure location with limited access in order to protect the privacy of the subjects.

ii. Who will have access to the research data, and how will access to the research data be controlled and monitored? (Please state the personnel who will have access to the study data eg. PI, Co-investigator, study coordinator.) * Click here for help

Only members of the Study Team will have access to the research data. The PC will password-protected and physically located in a controlled-access area of the COFM department.

All identifiable information of the subjects eg name, IC number on the laboratory investigation forms will NOT be written on questionnaires. The identifier in the questionnaire will be entered as a coded number. The link between the identifier and code number will be kept separately from the research data by a trusted thired party, ie the DSC Assistant Manager. This is only for the purpose of correctly matching the laboratory results, so that participants will receive the correct treatment for any diagnosed STI.

◉ No
○ Yes

Upon completion of the study, only non-identifiable data will be retained for writing up. All identifiable data will be destroyed 6 months after project completion.

○ Yes, the study team would send research data to the study sponsor

R2 Will any part of the study procedures be recorded on audiotape, film/video, or other electronic medium?

◉ No
○ Yes

This section shows the Principal Investigator's as well as Study Team Members' Curriculum Vitae. Please ensure that the information shown here is accurate and up to date.

If the PI or Study Team Member Currivulum Viate does not appear on the list, the team member needs to upload or update his/her CV, it could be done through his/her ROAM profile.

The DSRB will use the information contained here to assess the qualifications of the Principal Investigator and Study team members to carry out the Study as described in this Application.

No.	Study Site	Name	Study Role	CV
1	National Skin Centre	Dr PRIYA SEN	PI	Priya Sen CV 28 Aug 06.doc 30-Sep-201
2	National Skin Centre	Prof roy chan	Co-Investigator	CV full_Roy Chan_2 Jun 2011.doc 13-Jul-201
3	National Skin Centre	Dr Martin Chio	Collaborator	CV M Chio for ROAM Sep 2011.doc 29-Sep-201
4	National Skin Centre	Dr Hiok Hee Tan	Collaborator	THHCV2010 with pic .doc 30-Sep-201
5	NUS - Saw Swee Hock School of Public Health	A/Prof Mee Lian Wong	Site PI	cv1page wml 2012_ NIHA.doc 11-Jul-201
6	NUS - Saw Swee Hock School of Public Health	Prof David Koh	Co-Investigator	
7	NUS - Saw Swee Hock School of Public Health	Dr Judy Sng	Co-Investigator	

Your DSRB Application is now complete and ready for submission.

Principal Investigator's Declaration

I will not initiate this study until I have received approval notification from the DSRB and all applicable regulatory authorities.

I will not initiate any change in the study protocol without prior written approval from the DSRB, except when it is necessary to reduce or eliminate any immediate risks to the Research Participants. Thereafter, I will submit the proposed amendment to the DSRB and all applicable regulatory authorities for approval.

I will promptly report any unexpected or serious adverse events, unanticipated problems or incidents that may occur in the course of this study.

I will maintain all relevant documents and recognise that the DSRB staff and applicable regulatory authorities may inspect these records.

I understand that failure to comply with all applicable regulations, institutional and DSRB policies and requirements may result in the suspension or termination of this study.

I declare that there are no existing or potential conflicts of interest for any of the investigators participating in this study and their immediate family members. If there are, I have declared them in the relevant section of this application form.

By checking the "I agree" box, you confirm that you have read, understood and accept the Principal Investigator's Declaration

☑ I have read and agree to the above declaration.

Principal Investigator: PRIYA SEN
